# Association of Dietary Advanced Glycation End Products with Metabolic Syndrome in Young Mexican Adults

**DOI:** 10.3390/medicines5040128

**Published:** 2018-12-01

**Authors:** Kenny Mendoza-Herrera, Celia Aradillas-García, Miguel A. Mejía-Diaz, Jorge A. Alegría-Torres, Ma. Eugenia Garay-Sevilla, Claudia Luevano-Contreras

**Affiliations:** 1 Center for Nutrition and Health Research (CINyS), National Institute of Public Health (INSP), Cuernavaca, Morelos 62100, México; cinys26@insp.mx; 2 Coordination for the Innovation and Application of Science and Technology (CIACYT), Autonomous University of San Luis Potosí (UASLP), San Luis Potosí, San Luis Potosí 78210, Mexico; celia.uaslp@gmail.com; 3 Department of Nutrition, University of Central Mexico (UCEM), San Luis Potosí, San Luis Potosí 78250, Mexico; mdma9019@gmail.com; 4 Department of Pharmacy, University of Guanajuato (UG), Guanajuato, Guanajuato 36050, Mexico; giorgio_alegretto@hotmail.com; 5 Department of Medical Science, University of Guanajuato, León, Guanajuato 37320, Mexico; marugaray_2000@yahoo.com

**Keywords:** advanced glycation end products, diet, metabolic syndrome, young adults

## Abstract

**Background:** Consumption of dietary advanced glycation end products is linked to metabolic syndrome. The objective was to describe the association between dietary advanced glycation end products intake and metabolic syndrome in young Mexican adults. **Methods:** The present was a cross-sectional study in 126 Mexican adults 18–35 years old evaluating metabolic syndrome through the harmonized criteria. Macronutrients and dietary advanced glycation end products intake were estimated through three 24-hour dietary recalls and food composition tables. Association between metabolic syndrome and high advanced glycation end products intake (≥10,000 kU/day) was evaluated through three logistic regression models adjusted by sex, age, family history of cardiometabolic diseases and energy intake. **Results:** Subjects with a higher advanced glycation end products intake were more likely to have impaired fasting glucose (OR: 4.91, 95% CI 1.29–18.60, *p* < 0.05) and metabolic syndrome (OR: 2.67, 95% CI 0.96–7.44, *p* = 0.059) than those participants with low consumption of these products after adjustment of sex, age, family history of cardiovascular disease and energy intake. **Conclusions:** High intake of dietary advanced glycation end products was significantly associated with impaired fasting glucose and marginally with metabolic syndrome in young Mexican adults regardless of sex, age, family history of cardiovascular disease and energy intake.

## 1. Introduction

The metabolic syndrome (MS) is a cluster of risk factors for cardiovascular disease (CVD) and diabetes mellitus (DM) with the same pathophysiological basis: insulin resistance. It could be defined as the coexistence of the following components: obesity, impaired blood pressure, impaired glycemia and dyslipidemia [1]. In Mexico, a prevalence between 21.4% to 36.4% in individuals without DM have been reported [2,3]. According to the Mexican National Survey of Health and Nutrition 2006, the general prevalence of MS in adults without DM is 49.9%, with lower prevalence (27.1%) in 20–29 years old adults in comparison to 30–39 years old adults (44.5%) [4].

Physical inactivity and some dietary patterns especially the standard western diet which is high in fat, rich in meat and processed foods, could drive the pathophysiology of MS [5]. This type of diet is also a source of dietary advanced glycation end products (dAGEs). Dietary AGEs are a group of compounds formed by the Maillard reaction and autoxidation of fatty acids, among others [6,7]. According to several research groups foods rich in protein and fat had the highest amount of dAGEs and cereals had the lowest but this could increase depending on the processing. Factors such as cooking time, temperature, pH and humidity affect the rate of dAGEs formation. High cooking temperature for extended periods of time and an alkaline pH favor dAGEs formation [6,8]. In contrast, low pH and meals prepared with water or other liquid prevent dAGEs formation [9]. Therefore, foods that are fried, broiled, grilled or roasted would produce higher dAGEs than foods that are boiled, poached, stewed or steamed [6,7,10,11].

Advanced glycation end products (AGEs) are also formed endogenously by cellular metabolism. As well as in foods, several factors could increase the production of these substances endogenously, for instance, hyperglycemia [12] and oxidative stress [13]. High AGEs concentrations have been associated with normal aging, DM and its complications, CVD, arthritis, cancer, osteoporosis and Alzheimer disease [13]. A study in subjects without DM showed that the accumulation of AGEs in plasma and tissues is significantly associated with the individual risk factors of the MS, placing the individuals at high risk of developing DM and CVD [14]. dAGEs could act synergistically with endogenous AGEs and increase systematic AGEs load. AGEs could have an impact on health at least through two mechanisms: by accumulating in tissues, thus modifying proteins and by interacting with the receptor for AGEs (RAGE), thus increasing pro-inflammatory and pro-oxidant status [15].

Several studies in subjects with DM have shown consistently that dAGEs increases inflammation, oxidative stress and markers of endothelial dysfunction. For instance, correlations between dAGEs and high-sensitivity c-reactive protein [16], tumor necrosis factor alpha (TNF-*α*), vascular cell adhesion molecule 1, 8-isoprostane and with RAGE-mRNA [17] have been described. Additionally, Chao et al. compared healthy subjects (*n* = 74) and subjects with DM either with a low dAGE intake (*n* = 50) or with a high dAGE intake (*n* = 58). They found that subjects with DM and high dAGEs intake had elevated plasma levels of interleukin 1 alpha, TNF-*α*, 8-isoprostane, AGEs, hemoglobin A1c, LDL cholesterol, glycated low-density lipoprotein cholesterol and lower superoxide dismutase in comparison to healthy subjects [18]. Furthermore, a randomized trial in subjects with obesity 50 years and older showed that those with a low dAGEs intake had a reduction in the insulin resistance levels and in the waist circumference in comparison to those with high dAGEs intake. However, no similar results were observed for other MS components [19].

Only a few studies have evaluated the association of dAGEs and risk factors for MS in healthy subjects. Angoorani et al. showed that Iranian adults with higher intake of dAGEs were more likely to have abdominal obesity and hypertriglyceridemia. However, the study failed to show an association between individual components of the metabolic syndrome and dietary AGEs after adjusting for energy and macronutrients intake [20]. A similar cross-sectional study in American adolescents showed a significant association between high dAGEs intake and MS. Besides, an association was demonstrated with abdominal obesity and hypertriglyceridemia [21]. Given the potential impact of dAGEs intake on metabolic health, the objective of this study was to estimate the consumption of dAGEs in young Mexican adults and describe its association with risk factors for MS.

## 2. Materials and Methods

Students and administrative personnel from two Universities in San Luis Potosi, in North-Central Mexico, were invited to participate in a cross-sectional study between January 2013 and June of 2014. One hundred and forty volunteers 18 to 40 years old without a previous diagnosis of DM or CVD were recruited. However, only 126 subjects with completed questionnaires were included for final analysis. Before inclusion, all participants signed informed consent. The study was conducted according to the Declaration of Helsinki and the study was approved by the ethics committee of the University of Guanajuato (No. 2012-07, approval date: 8 November 2012). 

Standardized health personnel took all measurements following international standardized techniques. Weight, height and waist circumference were taken and body mass index (BMI) was calculated according to the formula (weight [Kg]/height [m]²). Participants blood pressure was measured twice with an interval of 30 s and they were asked if they had a previous high blood pressure diagnosis or if they were taking antihypertensive medications. A venous blood sample was obtained after 12 h fasting to measure glucose, triglycerides, total cholesterol and HDL cholesterol by conventional methods. 

The MS was evaluated according to the harmonizing criteria. Three or more of these criteria were considered: abdominal obesity (waist circumference ≥80 cm for women and ≥90 cm for men); high triglycerides (≥150 mg/dL); low HDL (≤50 mg/dL for women and ≤40 mg/dL for men); high systolic blood pressure (≥130 mmHg), and/or high diastolic blood pressure (≥85 mmHg) or use of antihypertensive medications; and impaired fasting glucose (≥100 mg/dL) [22].

In order to estimate usual dietary intake, each subject was interviewed by a dietitian and completed three 24-hour dietary recall in three different days of the week. During the interview, participants were asked for the place, time and amount of food and beverages consumed and particular emphasis was taken to record the cooking method and brand of food. Estimation of calories and macronutrients intake (proteins, lipids and carbohydrates) was carried out using the software Nutrikcal^®^. Disaggregation of dishes into single ingredients was done before entering the data on the software. When the reported amount was not found in Nutrikcal^®^ the website What’s In The Foods You Eat Search Tool (USDA, 2017) was used to decide the amount to be used in the software. The consumption of dAGEs was estimated by using a published food database with around 500 foods [6]. For quantification of total AGEs intake, the following steps were taken. First, data from the three recall questionnaires were entered into an Excel database (designed for this purpose, Microsoft, 2011). Second, the amount of dAGEs was calculated by multiplying the gram or milliliters of food consumed by the AGEs (KU) amount in 100 g of food. Finally, total dAGEs was determined by summing the intakes from each food. The publish database contains around 500 foods; therefore, some foods were not found in the database. These foods were calculated with averages from similar foods available. At least two researchers made all decisions and all final analyses were reviewed for any mistakes by an experienced dietitian. 

Besides, international dietary guidelines were used to evaluate nutrients intake [23]. High consumption of saturated fat and added-sugar were considered if it was higher than 10.0% of energy intake. High cholesterol intake was established if it was higher than 300.0 mg/day. Whereas, low consumption of polyunsaturated fat was considered as less than 6.0% of the energy intake. According to previous studies, a high dAGEs consumption was set as an intake higher or equal than 10,000 kU/AGEs/day [15].

For the statistical analysis, categorical variables were expressed as frequencies and proportions and the quantitative variables as means or medians and standard deviations (SD) or interquartile ranges, depending on their distribution. Comparison by MS or MS components status (presence or absence), was done with a Student t test or Mann–Whitney U test. Also, a χ^2^ test or Fisher exact test was used to compare proportions across categories of MS status. Besides, multivariate logistic regression was used to evaluate the association of the high dAGEs intake (main independent variable) with MS components (separately) and with MS (dependent variables). Three different models were developed. The model 1, adjusted by sex and age; the model 2 adjusted by sex, age and family history of cardiovascular risk (DM, hypertension and hypercholesterolemia) and the model 3, adjusted by the previous variables and by energy intake. Hypothesis testing was based on two-tailed tests and the statistical significance was set at *α* = 0.05. All the analyses were conducted on Stata version 14.1 (StataCorp LLC).

## 3. Results

### 3.1. Distribution of Clinical Characteristics by Metabolic Syndrome Status

For this study, 126 subjects with complete data were included for analyses. The median age was 23 (19, 30) years and 80.2% of the participants were women. The general prevalence of MS was 21.4%; 56.4% of the subjects had abdominal obesity, 38.1% had low HDL, 22.2% had hypertriglyceridemia, 16.7% had impaired fasting glucose and 15.9% had hypertension. Men had a significantly higher prevalence of MS (40.0%) in comparison to women (16.8%), *p* < 0.05. Subjects with MS had significantly higher age, blood pressure and fasting glucose levels when compared to those without the condition (*p* < 0.05), (Table 1).

Subjects with abdominal obesity had significantly higher systolic (112.0 vs. 103.0 mm Hg) and diastolic (73.3 vs. 67.7 mm Hg) blood pressure, as well as fasting glucose levels (91.0 vs. 86.0 mg/dL), in comparison to those with healthy waist circumference (*p* < 0.05). Similarly, subjects with low HDL and with hypertriglyceridemia had significantly higher fasting glucose levels (91.0 vs. 87.0 and 93.0 vs. 88.3 mg/dL [*p* < 0.05], respectively) than subjects without these conditions (Table 1).

### 3.2. Diet Characteristics and dAGEs Intake by Metabolic Syndrome Status

The energy, macronutrients and dAGEs intake by MS status is shown in Table 2. In the total group, the dAGEs intake was 10,240 kU/day and 52.4% of the participants reported a higher consumption (≥10,000 kU/day). Additionally, 39.2%, 37.6% and 16.0% of the participants had a high intake of cholesterol, saturated fat and added-sugar respectively; while 83.2% had a low polyunsaturated fat intake. Subjects with MS had a significantly higher intake of proteins and dAGEs when compared to those without the condition (344 vs. 284 g/day and 14,254 vs. 9,782 kU/day, *p* < 0.05). Similarly, a higher percentage of subjects with MS had a high cholesterol intake (63.0% vs. 32.7%, *p* < 0.05) and a high dAGEs intake (74.1% vs. 46.5% *p* < 0.05) when compared to participants without MS. There were no differences in energy, carbohydrates or fat intake between those with and without MS.

High cholesterol intake was more prevalent among subjects with abdominal obesity (48.6% vs. 27.3%), hypertriglyceridemia (59.3% vs. 33.7%) and hypertension (60.0% vs. 35.2%) than those without these conditions, *p* < 0.05. Moreover, the diet high in dAGEs was more prevalent in subjects with abdominal obesity (60.6% vs. 41.8%) and impaired fasting glucose (76.2% vs. 47.6%) when compared to those without these conditions, *p* < 0.05. There were no differences in energy, carbohydrates or fat intake between subjects with risk factors for MS and those without them (data is not shown).

### 3.3. Multivariate Analysis for Metabolic Syndrome according to High dAGEs Intake

Results for the association between dAGEs consumption (high vs. low intake) and risk factors for MS are shown in Table 3. There were no associations between high dAGEs intake and abdominal obesity, low HDL, hypertriglyceridemia and hypertension. After controlling by sex and age, a high dAGEs intake was associated with impaired fasting glucose (OR: 3.50, 95% CI 1.06, 11.46) and with MS status (OR: 2.79, 95% CI 1.03, 7.59), *p* < 0.05. The association remained after further adjustment by family history of cardiovascular risk and energy intake (*p* < 0.05) for impaired fasting glucose. On the other hand, once adjusting by family history of cardiovascular risk and energy intake, high dAGEs intake was marginally associated with MS (OR: 2.73, 95% CI 0.99, 7.54, *p* = 0.051; OR: 2.67, 95% CI 0.96, 7.44, *p* = 0.059).

## 4. Discussion

A positive association between high dAGEs intake and MS status was found in the study sample after adjusting by potential confounders. However, this association was marginal after further adjustment by family history of cardiovascular disease and energy intake. Moreover, high dAGEs was significantly associated with impaired fasting glucose regardless of sex, age, family history of cardiovascular disease and energy intake. These results are similar to a cross-sectional study in American adolescents where it was found a significant association between high dAGEs intake and MS [21]. In contrast, Angoorani et al. found an association between high dAGEs and abdominal obesity and hypertriglyceridemia, however, failed to show an association with MS after adjusting for energy and macronutrients intake [20].

Several researchers have found that high dAGEs intake is associated with higher concentration of circulating AGEs, inflammatory markers and increased insulin resistance [16,17,18,19]. Thus, that could explain the strong association between high dAGEs intake and impaired fasting glucose observed in the multivariate analysis of this study. It has been proposed that a high and sustained dAGEs consumption will increase the body AGEs load and decreased tissue AGE catabolism [19]. Therefore, long-term exposure to dAGEs could be a contributing factor in the chronic inflammation that underlies risk for the MS [24]. In this study, circulating AGEs were not measured; however, Uribarri et al. found a higher concentration of circulating AGEs in subjects with obesity and a risk factor for MS [24]. It is unclear in which extent exogenous or endogenous AGE contribute to the chronic oxidative stress and inflammation observed in MS. The higher plasma AGEs concentration in MS may be the result of several processes that could include a higher dAGEs intake; however, long-term clinical trials are needed to clarify the role of exogenous and endogenous AGEs have in the MS.

The dAGEs intake in this study was lower than the reported by Macias et al. in subjects from central Mexico with overweight or obesity (30–55 years old) (10,240 vs. 14,311 kU/day) [25]. Moreover, the dAGEs intake in this study was similar to that of an Iranian population (9686 kU/day) [20] but lower than the reported by healthy New Yorkers (14,700 kU/day) [24]. It is difficult to establish comparisons among these studies due to the study designs; however, the regional dietary patterns could explain in part these differences. New York has a food environment with a high density of fast food restaurants and where the preparation processes could increase the amount of dAGEs in foods. Likewise, this city has high accessibility to, availability and consumption of ultra-processed foods, which have a high quantity of dietary AGEs [6,26,27].

In this study, subjects with abdominal obesity and MS had a higher proportion of high cholesterol in their diet, a higher intake of proteins and a high dAGEs intake. These results could be partially explained by the fact that cholesterol and protein-rich foods are also higher in dAGEs, for instance, red meat [28]. The red meat has a high content of specific precursors of dAGEs formation, such as reactive amino lipids, fructose and glucose-6-phosphate (the last two are reducing carbohydrates) which could promote dAGEs formation [6,7].

One of the limitations of our study is the lack of information on smoking and physical activity, both involved in the pathophysiology of hyperglycemia, MS components and serum AGEs [29,30,31]. Hence, we cannot discard a possible confounding effect of these variables in the association between dAGEs intake and MS and its components. Another limitation was our reduced sample size, which could have attenuated the association between dAGEs and MS and its risk factors. Therefore, a post hoc power calculation based on a χ^2^ test was carried out. A given α value of 0.05 and a sample size of 126 were considered. The difference between probabilities of having MS given the two categories of dAGEs consumption (≥10,000 kU/day and <10,000 kU/day) according to multivariate model 3, was used as the given effect size [0.272 − 0.134 = 0.138]. The result was a β = 0.82 (data is not shown), therefore the association of dAGEs intake and MS could be attenuated and a larger sample size could be recommended.

To the best of our knowledge, this is the first study specifically designed to evaluate dAGEs intake and its association with MS. In order to estimate dAGEs, each subject completed a 24-hour dietary recall in three different days of the week emphasizing cooking methods and food brands. Angoorani et al. and Saha et al. used dietary questionnaires recorded for other purposes [20,21].

## 5. Conclusions

In conclusion, high dAGEs intake was associated with impaired fasting glucose in young Mexican adults, independently of sex, gender, family history of cardiometabolic diseases and energy intake. Furthermore, consumption of dAGEs was associated with MS, however this association was marginal after further adjustment by family history of cardiovascular disease and energy intake. Hence larger population-based studies evaluating lifestyle profiles and the consumption of these products are needed, in order to provide a better understanding of the contribution of dAGEs intake to metabolic health in the Mexican population.

## Figures and Tables

**Table 1:** Prevalence of metabolic syndrome risk factors and distribution of clinical characteristics by metabolic syndrome status.

Clinical and Metabolic Characteristics	Abdominal Obesity		Low HDL-C		High Triglycerides		Impaired Fasting Glucose		Impaired Blood Pressure		Metabolic Syndrome	
	**Yes**, %	**No**, %	**Yes**, %	**No**, %	**Yes**, %	**No**, %	**Yes**, %	**No**, %	**Yes**, %	**No**, %	**Yes**, %	**No**, %
**Total (*n* = 126)**	56.4	43.7	38.1	61.9	22.2	77.8	16.7	83.3	15.9	84.1	21.4	78.6
**Age** **(y)**	27.0 (13.0)	20.0 * (6.0)	20.0 (10.5)	25.0 (11.0)	28.0 (10.5)	21.0 * (9.0)	32.0 (8.0)	21.0 * (9.0)	24.5 (8.0)	22.0 (11.0)	29.0 (12.0)	21.0 * (9.0)
**Systolic blood pressure (mmHg)**	112.0 (16.0)	103.0 * (16.0)	110.0 (18.5)	110.0 (20.0)	115.5 (23.0)	110.0 (20.0)	122.0 (10.0)	110.0 * (20.0)	130.0 (6.5)	110.0 * (17.0)	123.0 (21.0)	110.0 * (18.0)
**Diastolic blood pressure (mmHg)**	73.3 (9.9)	67.7 * (8.12)	72.7 (9.4)	69.7 (9.6)	74.4 (8.9)	69.8 * (9.6)	75.7 (10.0)	69.9 * (9.2)	84.1 (7.2)	68.3 * (7.7)	77.6 (10.4)	69.0 * (8.5)
**Glucose (mg/dL)**	91.0 (13.6)	86.0 * (10.0)	91.0 (11.8)	87.0 * (11.0)	93.0 (18.9)	88.3 * (11.0)	108.7 (10.7)	87.5 * (7.8)	94.0 (19.0)	89.0 (11.0)	101.0 (18.8)	88.0 * (8.0)
**Cholesterol (mg/dL)**	148.0 (57.3)	159.0 (38.1)	154.5 (57.8)	152.4 (45.1)	156.9 (63.5)	154.0 (53.6)	148.0 (58.0)	155.0 (51.2)	153.1 (49.8)	154.0 (53.6)	155.0 (58.0)	154.0 (52.1)
**HDL-C (mg/dL)**	49.0 (21.0)	54.0 * (19.0)	41.1 (11.0)	60.4 * (17.5)	47.6 (15.1)	51.4 (21.1)	47.6 (11.9)	51.0 (20.1)	48.9 (18.2)	51.0 (21.0)	44.0 (14.4)	52.8 * (19.8)
**Triglycerides (mg/dL)**	122.2 (68.9)	92.0 * (56.8)	118.3 (69.5)	97.8 (64.8)	206.5 (84.3)	92.5 * (48.3)	154.8 (58.0)	100.6 * (63.1)	121.5 (74.9)	105.1 (67.0)	160.0 (81.0)	93.0 * (51.5)

Data are shown as means and standard deviations for variables with normal distribution or as medians and interquartile range for variables without a normal distribution. * This indicates a statistically significant difference (*p* < 0.05) from a χ^2^ test or a Fisher exact test (proportion comparison or for contingency tables of more than 2 categories)/Student *t*-test or a Mann–Whitney U test between positive and negative categories of metabolic syndrome components and metabolic syndrome. HDL-C: high-density lipoprotein cholesterol.

**Table 2:** Diet characteristics and dAGEs intake by metabolic syndrome status.

Dietary Characteristics		Metabolic Syndrome	
	(*n* = 126)	Yes	No
	Total		
**Energy (Kcal)**	1844 (762)	1974 (1131)	1790 (702)
**Carbohydrates (Kcal)**	908 (516)	1008 (620)	880 (488)
**Proteins (Kcal)**	300 (116)	344 (144)	284 * (100)
**Fats (Kcal)**	621 (351)	639 (522)	603 (378)
**dAGES intake (kU/day)**	10,240 (6855)	14,254 (8162)	9782 * (6832)
**High saturated fats diet (≥10.0 from energy) (%)**	37.6 (29.1, 46.1)	37.0 (18.8, 55.3)	37.8 (28.2, 47.4)
**Low polyunsaturated fats diet (<6.0% from energy) (%)**	83.2 (76.6, 89.8)	85.2 (71.8, 98.6)	82.7 (75.2, 90.1)
**High cholesterol diet (≥300.0 mg/day) (%)**	39.2 (30.6, 47.8)	63.0 (44.7, 81.2)	32.7 * (23.4, 41.9)
**High added-sugar diet (≥10.0% of energy) (%)**	16.0 (9.6, 22.4)	11.1 (−0.7, 23.0)	17.3 (9.9, 24.8)
**High dAGEs intake (≥10,000 kU) (%)**	52.4 (43.7, 61.1)	74.1 (57.5, 90.6)	46.5 * (36.6, 56.3)

Data of energy, carbohydrates, proteins, fats (Kcal) and dAGEs intake (kU/day) are shown as medians and interquartile range. Data of diet high in saturated fats, polyunsaturated fats, cholesterol, added-sugar and dAGEs are shown as a percentage and 95%CI. * This indicates a statistically significant difference (*p* < 0.05) from a χ^2^ test or a Fisher exact test (proportion comparison) or Mann–Whitney U test between positive and negative categories of metabolic syndrome components. dAGEs: dietary advanced glycation end products.

**Table 3:** Multivariate analysis for metabolic syndrome and its components according to high dAGEs intake.

dAGEs Intake (reference <10,000 kU/day)	Model 1 ^a^		Model 2 ^b^		Model 3 ^c^	
***Abdominal obesity***	**OR**	**95% CI**	**OR**	**95% CI**	**OR**	**95% CI**
*High (≥10,000 kU/day)*	2.07	(0.94, 4.55)	1.91	(0.84, 4.30)	1.81	(0.78, 4.18)
***Low HDL-C***						
*High (≥10,000 kU/day)*	1.65	(0.78, 3.51)	1.69	(0.77, 3.69)	1.62	(0.72, 3.62)
***Hypertriglyceridemia***						
*High (≥10,000 kU/day)*	1.62	(0.65, 4.04)	1.54	(0.59, 4.00)	1.34	(0.50, 3.60)
***Impaired Fasting Glucose***						
*High (≥10,000 kU/day)*	3.50 *	(1.06, 11.46)	4.86 *	(1.29, 18.31)	4.91 *	(1.29, 18.60)
***Hypertension***						
*High (≥10,000 kU/day)*	0.92	(0.30, 2.81)	0.98	(0.31, 3.07)	1.25	(0.38, 4.15)
***Metabolic Syndrome***						
*High (≥10,000 kU/day)*	2.79 *	(1.03, 7.59)	2.73	(0.99, 7.54)	2.67	(0.96, 7.44)

* This indicates a statistically significant OR in multivariate models (*p* < 0.05). ^a^ Logistic regression model adjusted by age and sex. ^b^ Logistic regression model adjusted by age, sex and family history of cardiovascular risk (diabetes, hypertension and hypercholesterolemia). ^c^ Logistic regression model adjusted by age, sex, family history of cardiovascular risk and energy intake. dAGEs: dietary advanced glycation end products. HDL-C: high-density lipoprotein cholesterol.

## References

[1] Reaven G.M. (1988). Banting lecture 1988. Role of insulin resistance in human disease. Diabetes.

[2] Aguilar-Salinas C.A., Rojas R., Gómez-Pérez F.J., Valles V., Ríos-Torres J.M., Franco A., Olaiz G., Rull J.A., Sepúlveda J. (2004). High prevalence of metabolic syndrome in Mexico. Arch. Med. Res..

[3] Echavarría-Pinto M., Hernández-Lomelí A., Alcocer-Gamba M.A., Morales-Flores H., Vázquez-Mellado A. (2006). Síndrome metabólico en adultos de 20 a 40 años en una comunidad rural mexicana. Rev. Med. Inst. Mex. Seguro Soc..

[4] Pedroza-Tobias A., Trejo-Valdivia B., Sanchez-Romero L.M., Barquera S. (2014). Classification of metabolic syndrome according to lipid alterations: Analysis from the Mexican National Health and Nutrition Survey 2006. BMC Public Health.

[5] Lutsey P.L., Steffen L.M., Stevens J. (2008). Dietary intake and the development of the metabolic syndrome: The Atherosclerosis Risk in Communities study. Circulation.

[6] Uribarri J., Woodruff S., Goodman S., Cai W., Chen X., Pyzik R., Yong A., Striker G.E., Vlassara H. (2010). Advanced glycation end products in foods and a practical guide to their reduction in the diet. J. Am. Diet. Assoc..

[7] Chao P.-C., Hsu C.-C., Yin M.-C. (2009). Analysis of glycative products in sauces and sauce-treated foods. Food Chem..

[8] Luevano-Contreras C., Garay-Sevilla M.E., Preciado-Puga M., Chapman-Novakofski K.M. (2013). The relationship between dietary advanced glycation end products and indicators of diabetes severity in Mexicans and non-Hispanic whites: A pilot study. Int. J. Food Sci. Nutr..

[9] Poulsen M.W., Hedegaard R.V., Andersen J.M., de Courten B., Bügel S., Nielsen J., Skibsted L.H., Dragsted L.O. (2013). Advanced glycation endproducts in food and their effects on health. Food Chem. Toxicol..

[10] Hull G.L.J., Woodside J.V., Ames J.M., Cuskelly G.J. (2012). Nε-(carboxymethyl)lysine content of foods commonly consumed in a Western style diet. Food Chem..

[11] Scheijen J.L.J.M., Clevers E., Engelen L., Dagnelie P.C., Brouns F., Stehouwer C.D.A., Schalkwijk C.G. (2016). Analysis of advanced glycation endproducts in selected food items by ultra-performance liquid chromatography tandem mass spectrometry: Presentation of a dietary AGE database. Food Chem..

[12] Vlassara H., Uribarri J. (2014). Advanced glycation end products (AGE) and diabetes: Cause, effect, or both?. Curr. Diabetes Rep..

[13] Uribarri J., del Castillo M.D., de la Maza M.P., Filip R., Gugliucci A., Luevano-Contreras C., Macías-Cervantes M.H., Markowicz Bastos D.H., Medrano A., Menini T. (2015). Dietary advanced glycation end products and their role in health and disease. Adv. Nutr..

[14] Sebekova K., Krivosikova Z., Gajdos M. (2014). Total plasma Nepsilon-(carboxymethyl)lysine and sRAGE levels are inversely associated with a number of metabolic syndrome risk factors in non-diabetic young-to-middle-aged medication-free subjects. Clin. Chem. Lab. Med..

[15] Luevano-Contreras C., Gomez-Ojeda A., Macias-Cervantes M.H., Garay-Sevilla M.E. (2017). Dietary Advanced Glycation End Products and Cardiometabolic Risk. Curr. Diabates Rep..

[16] Uribarri J., Cai W., Peppa M., Goodman S., Ferrucci L., Striker G., Vlassara H. (2007). Circulating glycotoxins and dietary advanced glycation endproducts: Two links to inflammatory response, oxidative stress, and aging. J. Gerontol. A Biol. Sci. Med. Sci..

[17] Uribarri J., Cai W., Pyzik R., Goodman S., Chen X., Zhu L., Ramdas M., Striker G.E., Vlassara H. (2014). Suppression of native defense mechanisms, SIRT1 and PPARgamma, by dietary glycoxidants precedes disease in adult humans; relevance to lifestyle-engendered chronic diseases. Amino Acids.

[18] Chao P.C., Huang C.N., Hsu C.C., Yin M.C., Guo Y.R. (2010). Association of dietary AGEs with circulating AGEs, glycated LDL, IL-1alpha and MCP-1 levels in type 2 diabetic patients. Eur. J. Nutr..

[19] Vlassara H., Cai W., Tripp E., Pyzik R., Yee K., Goldberg L., Tansman L., Chen X., Mani V., Fayad Z.A. (2016). Oral AGE restriction ameliorates insulin resistance in obese individuals with the metabolic syndrome: A randomised controlled trial. Diabetologia.

[20] Angoorani P., Ejtahed H.-S., Mirmiran P., Mirzaei S., Azizi F. (2016). Dietary consumption of advanced glycation end products and risk of metabolic syndrome. Int. J. Food Sci. Nutr..

[21] Saha A., Poojary P., Chan L., Chauhan K., Nadkarni G., Coca S., Uribarri J. (2017). Increased odds of metabolic syndrome with consumption of high dietary advanced glycation end products in adolescents. Diabetes MeTable.

[22] Alberti K.G., Eckel R.H., Grundy S.M., Zimmet P.Z., Cleeman J.I., Donato K.A., Fruchart J.C., James W.P., Loria C.M., Smith S.C. (2009). Harmonizing the metabolic syndrome: A joint interim statement of the International Diabetes Federation Task Force on Epidemiology and Prevention; National Heart, Lung, and Blood Institute; American Heart Association; World Heart Federation; International Atherosclerosis Society; and International Association for the Study of Obesity. Circulation.

[23] FAO, World Health Organization (2003). Diet, Nutrition and the Prevention of Chronic Diseases: Report of a Joint WH. http://www.who.int/dietphysicalactivity/publications/trs916/en/.

[24] Uribarri J., Cai W., Woodward M., Tripp E., Goldberg L., Pyzik R., Yee K., Tansman L., Chen X., Mani V. (2015). Elevated serum advanced glycation endproducts in obese indicate risk for the metabolic syndrome: A link between healthy and unhealthy obesity?. J. Clin. Endocrinol. MeTable.

[25] Macías-Cervantes M.H., Rodríguez-Soto J.M.D., Uribarri J., Díaz-Cisneros F.J., Cai W., Garay-Sevilla M.E. (2015). Effect of an advanced glycation end product-restricted diet and exercise on metabolic parameters in adult overweight men. Nutrition.

[26] Sharma C., Kaur A., Thind S., Singh B., Raina S. (2015). Advanced glycation end-products (AGEs): An emerging concern for processed food industries. J. Food Sci. Technol..

[27] Steele E.M., Baraldi L.G., da Costa Louzada M.L., Moubarac J.-C., Mozaffarian D., Monteiro C.A. (2016). Ultra-processed foods and added sugars in the US diet: Evidence from a nationally representative cross-sectional study. BMJ Open..

[28] Rohrmann S., Linseisen J. (2016). Processed meat: The real villain?. Proc. Nutr. Soc..

[29] Cerami C., Founds H., Nicholl I., Mitsuhashi T., Giordano D., Vanpatten S., Lee A., Al-Abed Y., Vlassara H., Bucala R. (1997). Tobacco smoke is a source of toxic reactive glycation products. Proc. Natl. Acad. Sci. USA.

[30] Umpierre D., Ribeiro P.A., Kramer C.K., Leitão C.B., Zucatti A.T., Azevedo M.J., Gross J.L., Ribeiro J.P., Schaan B.D. (2011). Physical activity advice only or structured exercise training and association with HbA1c levels in type 2 diabetes: A systematic review and meta-analysis. JAMA.

[31] Sun K., Liu J., Ning G. (2012). Active smoking and risk of metabolic syndrome: A meta-analysis of prospective studies. PLoS ONE.

